# E-cadherin and **α**-, **β**- and **γ**-catenin expression in prostate cancers: correlation with tumour invasion

**DOI:** 10.1038/sj.bjc.6690299

**Published:** 1999-04

**Authors:** N Morita, H Uemura, K Tsumatani, M Cho, Y Hirao, E Okajima, N Konishi, Y Hiasa

**Affiliations:** Department of Urology, 2nd Department of Pathology, Nara Medical University, Shijo-cho 840, Kashihara, Nara, 634, Japan

**Keywords:** E-cadherin, catenins, prostate cancer, immunohistochemistry, tumour invasion

## Abstract

The E-cadherin–catenin complex plays an important role in establishing and maintaining intercellular connections and morphogenesis and reduced expression of its constituent molecules is associated with invasion and metastasis. In the present study, we examined E-cadherin and α-, β- and γ-catenin levels in tumour tissues obtained by radical prostatectomy in order to investigate the relationship with histopathological tumour invasion. Immunohistochemical findings for 45 prostate cancer specimens demonstrated aberrant expression of each molecule to be associated with dedifferentiation and, in addition, alteration of staining patterns for the three types of catenin was significantly correlated with capsular but not lymphatic or vascular invasion. The data thus suggest that three types of catenin may be useful predictive markers for biological aggressiveness of prostate cancer. © 1999 Cancer Research Campaign


*British Journal of Cancer* (1999) **79**(11/12), 1879–1883
© 1999 Cancer Research Campaign
Article no. bjoc.1998.0299

### E-cadherin and α-, β- and γ-catenin expression in prostate cancers: correlation with tumour invasion

**N Morita[1], H Uemura[1], K Tsumatani[1], M Cho[1], Y Hirao[1], E Okajima[1], N Konishi[2] and Y Hiasa[2]**

[1]Department of Urology and [2]2nd Department of Pathology, Nara Medical University, Shijo-cho 840, Kashihara, Nara 634, Japan

**Summary** The E-cadherin–catenin complex plays an important role in establishing and maintaining intercellular connections and morphogenesis and reduced expression of its constituent molecules is associated with invasion and metastasis. In the present study, we examined E-cadherin and α-, β- and γ-catenin levels in tumour tissues obtained by radical prostatectomy in order to investigate the relationship with histopathological tumour invasion. Immunohistochemical findings for 45 prostate cancer specimens demonstrated aberrant expression of each molecule to be associated with dedifferentiation and, in addition, alteration of staining patterns for the three types of catenin was significantly correlated with capsular but not lymphatic or vascular invasion. The data thus suggest that three types of catenin may be useful predictive markers for biological aggressiveness of prostate cancer.

**Keywords:** E-cadherin; catenins; prostate cancer; immunohistochemistry; tumour invasion

In the United States and the European countries, prostate cancer has become the second and third leading cause of cancer death in men, respectively (Boring et al, 1993; Black et al, 1997). Associated mortality in Japan has also increased in recent years with the Westernization of dietary habits (Tominaga and Kuroishi, 1997). The continuing controversy as to whether PSA-based screening and early detection of prostate cancer may contribute to decreasing its morbidity and mortality (Hall, 1996; Smart, 1997) is partly due to the difficulty in predicting biological potential. Although clinical stage and grade have been widely utilized as prognostic factors in prostate cancer, their value appears limited. At every stage, both good and poor clinical courses are found (Grayhack and Assimos, 1983). Furthermore, while the extremes of tumour grade, e.g. well and poorly differentiated, are useful in formulating a probable prognosis for patients (Grayhack and Assimos, 1983), the majority of patients have moderately differentiated or Gleason's grade 5–7 tumours (Grayhack and Assimos, 1983; Carter and Coffey, 1989). Therefore, identification of new markers which might accurately reflect biological aggressiveness of prostate cancers remains a high priority.

E-cadherin is a $Ca^{2+}$-dependent intercellular adhesion molecule in epithelial cells which plays an important role in establishing and maintaining intercellular connections and morphogenesis (Takeichi, 1991). This transmembrane glycoprotein forms a complex with catenins, which can be divided into α, β and γ on the basis of their electrophoretic mobilities (Ozawa et al, 1989). E-cadherin binds directly to either β-catenin or γ-catenin, whereas α-catenin links the bound E-cadherin complex to the actin cytoskeleton (Hinck et al, 1994).

It has been suggested that dysfunctional E-cadherin-mediated cell adhesion is associated with tumour invasion and metastasis. In vitro studies have shown that loss of E-cadherin expression is correlated with an invasive phenotype (Behrens et al, 1989; Vleminckx et al, 1991; Bussemakers et al, 1992) and aberrant E-cadherin expression is associated with poor differentiation and an invasive phenotype in several human cancers (Umbas et al, 1994; Syrigos et al, 1995; Siitonen et al, 1996; Wasielewski et al, 1997). Similarily, aberrant expression of membrane-bound α- and β-catenin has been observed in cancer cell lines and tumour tissues (Kadowaki et al, 1994; Matsui et al, 1994; Ochiai et al, 1994; Pierceall et al, 1995; Rimm et al, 1995). Downregulation of these catenins seems to be associated with dysfunction of E-cadherin-mediated cell adhesion and increase of the metastatic potential of cancer cells (Kadowaki et al, 1994; Matsui et al, 1994; Ochiai et al, 1994; Pierceall et al, 1995; Rimm et al, 1995). Recent studies have further revealed that free cytoplasmic β-catenin behaves as an oncoprotein in the APC-β-catenin-Tcf pathway (Korinek et al, 1997; Morin et al, 1997). It remains unresolved whether membrane-bound β-catenin in the cadherin–catenin complex may affect the free cytoplasmic β-catenin pool (Nakamura, 1997).

Coexpression of E-cadherin and α-, β- and γ-catenin has been studied in bladder and gastric cancers (Shimazui et al, 1996; Jawhari et al, 1997), but not to our knowledge in prostate neoplasms. Therefore the present immunohistochemical examination of E-cadherin and α, β, γ-catenin levels in radical prostatectomy specimens from 45 prostate cancer patients not undergoing any preoperative treatment was performed. Particular attention was concentrated on the correlation between expression pattern and types of histopathologically defined tumour invasion.

### MATERIALS AND METHODS

### Tissue specimen

Tissue specimens were obtained from 45 patients with prostate cancer who underwent radical prostatectomy in Nara Medical University Hospital or its affiliated hospitals between January

*Received 15 May 1998*
*Revised 24 August 1998*
*Accepted 29 September 1998*

*Correspondence to:* H Uemura

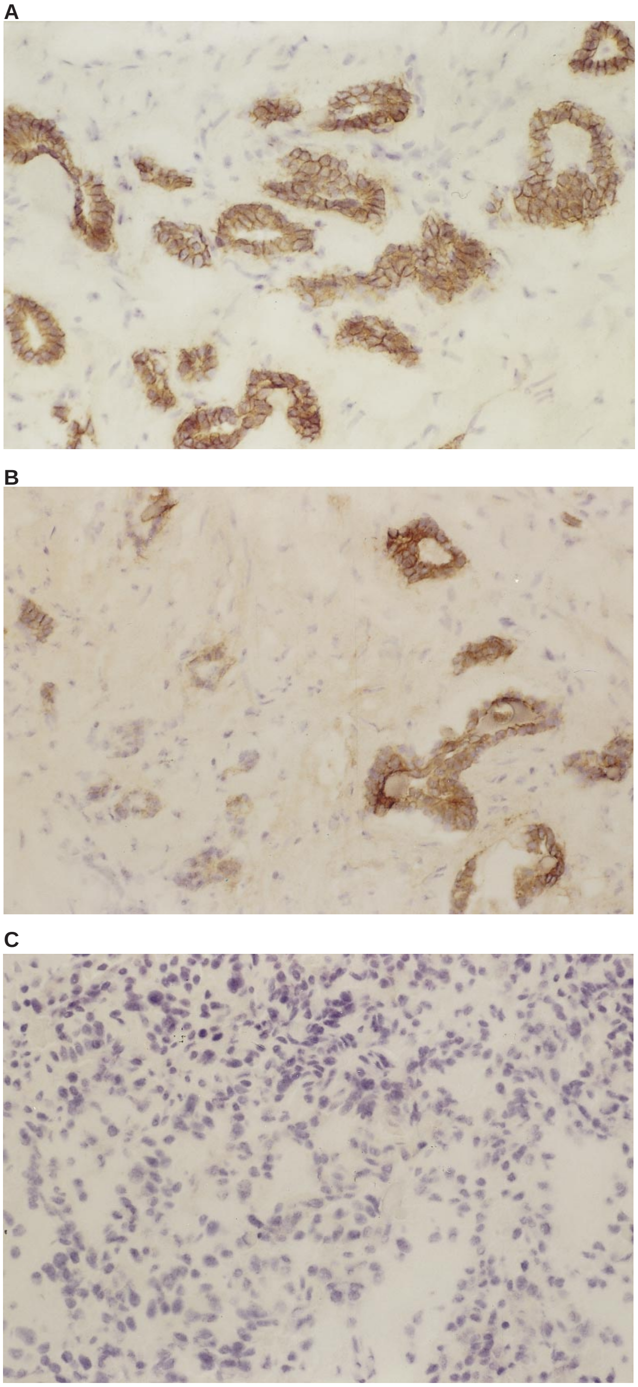

**Figure 1(A)** Normal immunostaining for E-cadherin in a prostatic adenocarcinoma (Gleason score 6), magnification × 200.
**(B)** Heterogeneous immunostaining for α-catenin in a prostatic adenocarcinoma (Gleason score 7), magnification × 200.
**(C)** Completely negative immunostaining for β-catenin in a prostatic adenocarcinoma (Gleason score 9), magnification × 200

1994 and June 1997. The mean age of patients was 67.2 years, ranging from 54 to 76 years. Bone scan and CT scan of the pelvis did not reveal any metastasis and none of the patients received treatments such as androgen-deprivation therapy, chemotherapy or radiation therapy before surgery.

Each specimen was serially cut at 5-mm intervals vertically to the urethra. One slice of the whole prostate was fixed in 10% neutral buffered formalin, embedded in paraffin, and sectioned at 5 μm and the remaining slices were frozen at –70°C for subsequent immunohistochemistry. Sections were stained with haematoxylin and eosin (HE) for mapping of cancer-affected areas. The histological differentiation of prostate cancer was determined according to the Gleason system (Gleason, 1977). Histopathological diagnosis was made by one pathologist (NK) to ensure consistency.

### Immunohistochemistry

Based on HE evaluation of formalin-fixed tissue, regions were selected for immunohistochemistry from frozen specimens. The seminal vesicles were excluded from immunohistochemical analysis. Immunostaining for E-cadherin and the three types of catenin was performed using a standard avidin–biotin immunoperoxidase technique. Antibodies used were HECD-1 (1:1000) for E-cadherin (Takara, Shiga, Japan), anti-α-catenin (1:100), anti-β-catenin (1:100) and anti-γ-catenin (1:100) (Transduction Laboratories, Kentucky, USA). Briefly, 5-μm frozen sections were air-dried and fixed with 3% paraformaldehyde. After endogeneous peroxidase was blocked using 0.3% hydrogen peroxidase in methanol, non-specific staining was eliminated by 30 min of incubation with normal horse serum. The sections were incubated at 37°C with primary antibodies for 60 min, biotin-labelled secondary antibody for 30 min and ABC complex for 30 min (Vectastatin ABC kit, Vector Laboratories, Inc., CA, USA). Peroxidase reaction was visualized with a solution of DAB (diaminobenzidine tetrahydrochloride) supplemented with 0.01% hydrogen peroxidase. The sections were counterstained with haematoxylin, dehydrated and mounted.

Evaluation of immunostaining was done by two independent observers (NM and MC) as follows: uniformly positive staining was regarded as normal, while uniformly negative or heterogeneous (mixed populations of positive and negative cells) staining as aberrant (Figure 1). Areas of benign prostatic hyperplasia or normal prostatic glands adjacent to prostate cancers were used as internal positive controls.

### Statistical analyses

Correlation between expression of each molecule and clinicopathological parameters was evaluated using the $\chi^2$ test. Probability values < 0.05 were considered significant.

### RESULTS

Histopathological findings for all cases are summarized in Table 1. Very few lesions could be categorized as well-differentiated (Gleason score 3–4) type (3/45, 7%), with the remainder being equally divided into moderately differentiated (Gleason score 5–7) and poorly differentiated (Gleason score 8–10) types (22/45, 49% and 20/45, 44%, respectively). Capsular invasion was apparent in almost half of all cases (22/45, 49%) with most of them being extracapsular (16/22, 73%). Lymphatic duct were frequently invaded (31/45, 69%), while vascular invasion was not frequent (8/45, 18%). Seminal vesicle invasion and lymph-node metastasis were also uncommon events (8/45, 18% and 3/45, 7%, respectively) (data not shown).

© Cancer Research Campaign 1999

**Table 1** Histopathological findings and E-cadherin and catenins expression in all prostate cancers

| Case | Gleason score | Capsular invasion | Lymphatic invasion | Vascular invasion | Expression | | | |
|---|---|---|---|---|---|---|---|---|
| | | | | | E-cadherin | α-catenin | β-catenin | γ-catenin |
| 1 | 3 | (–) | (–) | (–) | N | N | N | N |
| 2 | 3 | (–) | (–) | (–) | N | N | N | N |
| 3 | 4 | (–) | (–) | (–) | N | N | N | N |
| 4 | 5 | (+) | (+) | (–) | A | A | A | A |
| 5 | 5 | (+)[a] | (+) | (–) | A | A | A | A |
| 6 | 5 | (+)[a] | (+) | (–) | N | N | N | N |
| 7 | 5 | (+)[a] | (+) | (–) | N | N | N | N |
| 8 | 5 | (–) | (–) | (–) | N | N | N | N |
| 9 | 6 | (+)[a] | (+) | (+) | N | N | N | N |
| 10 | 6 | (+) | (–) | (–) | A | A | A | A |
| 11 | 6 | (–) | (+) | (–) | N | N | N | N |
| 12 | 6 | (–) | (+) | (–) | N | N | N | N |
| 13 | 6 | (–) | (+) | (+) | N | N | N | N |
| 14 | 6 | (–) | (–) | (–) | N | N | N | N |
| 15 | 7 | (+) | (+) | (–) | A | A | A | N |
| 16 | 7 | (+)[a] | (+) | (–) | N | N | N | N |
| 17 | 7 | (+)[a] | (+) | (+) | N | N | N | N |
| 18 | 7 | (+) | (+) | (+) | A | A | A | A |
| 19 | 7 | (–) | (+) | (–) | N | N | N | N |
| 20 | 7 | (–) | (+) | (–) | N | N | N | N |
| 21 | 7 | (–) | (+) | (–) | N | A | A | N |
| 22 | 7 | (–) | (+) | (–) | N | N | N | N |
| 23 | 7 | (–) | (–) | (–) | N | N | N | N |
| 24 | 7 | (–) | (–) | (–) | A | A | N | N |
| 25 | 7 | (–) | (–) | (–) | A | A | N | N |
| 26 | 8 | (+)[a] | (+) | (–) | N | N | N | N |
| 27 | 8 | (+)[a] | (+) | (–) | N | A | A | N |
| 28 | 8 | (+)[a] | (+) | (–) | A | A | A | A |
| 29 | 8 | (+)[a] | (+) | (+) | A | A | A | N |
| 30 | 8 | (+)[a] | (+) | (–) | N | N | A | A |
| 31 | 8 | (+)[a] | (+) | (+) | A | A | N | N |
| 32 | 8 | (–) | (+) | (–) | N | N | N | N |
| 33 | 8 | (–) | (–) | (–) | A | A | A | A |
| 34 | 9 | (+)[a] | (+) | (+) | A | A | A | N |
| 35 | 9 | (+)[a] | (+) | (+) | A | A | A | A |
| 36 | 9 | (+)[a] | (+) | (–) | A | A | A | A |
| 37 | 9 | (+)[a] | (+) | (–) | N | A | A | A |
| 38 | 9 | (+) | (–) | (–) | A | A | N | A |
| 39 | 9 | (–) | (+) | (–) | A | A | A | A |
| 40 | 9 | (–) | (+) | (–) | A | N | N | N |
| 41 | 9 | (–) | (+) | (–) | N | N | N | N |
| 42 | 9 | (–) | (–) | (–) | A | A | A | A |
| 43 | 9 | (–) | (–) | (–) | A | A | N | N |
| 44 | 9 | (–) | (–) | (–) | A | N | A | N |
| 45 | 10 | (+) | (+) | (–) | A | A | A | A |

[a]Capsular penetration. N, normal expression; A, aberrant expression.

**Table 2** Relationship between E-cadherin and catenin expression

| E-cadherin expression | No. | Catenin expression | | | Significance |
|---|---|---|---|---|---|
| | | N[a] | N+A[b] | A[c] | |
| N | 24 | 20 | 3 | 1 | $\chi^2 = 28.45$ |
| A | 21 | 1 | 9 | 11 | $P < 0.0001$ |

N, normal expression; A, aberrant expression. [a]α-, β- and γ-catenin all normally expressed. [b]α-, β- and γ-catenin aberrantly expressed singly or in pairs.[c]α-, β- and γ-catenin all aberrantly expressed.

Data for expression patterns of E-cadherin and the three types of catenin are also summarized in Table 1. The frequencies of normal and aberrant expression were, respectively, 24 and 21 for E-cadherin, 23 and 22 for α-catenin, 26 and 19 for β-catenin, and 31 and 14 for γ-catenin. Invading groups of cancer cells generally showed the same expression pattern as primary nests. No correlation with the preoperative PSA value was evident (data not shown). Expression of E-cadherin was consistent with those of catenins in 31 cases (69%). Of the other 14 (31%), four showed normal E-cadherin with aberrant expression of at least one type of catenin and 10

**Table 3** Relationship between expression of E-cadherin and catenins and the Gleason score

| Gleason score | E-cadherin | | | α-catenin | | | β-catenin | | | γ-catenin | | |
|---|---|---|---|---|---|---|---|---|---|---|---|---|
| | N | A | Significance | N | A | Significance | N | A | Significance | N | A | Significance |
| 3–4 | 3 | 0 | | 3 | 0 | | 3 | 0 | | 3 | 0 | |
| 5–7 | 15 | 7 | $\chi^2 = 8.95$ | 14 | 8 | $\chi^2 = 7.82$ | 16 | 6 | $\chi^2 = 8.46$ | 18 | 4 | $\chi^2 = 6.40$ |
| 8–10 | 6 | 14 | $P = 0.011$ | 6 | 14 | $P = 0.020$ | 7 | 13 | $P = 0.015$ | 10 | 10 | $P = 0.041$ |

N, normal expression; A, aberrant expression.

**Table 4** Relationship between expression of E-cadherin and catenins and capsular invasion

| | E-cadherin | α-catenin | β-catenin | γ-catenin |
|---|---|---|---|---|
| All cases ($n = 45$) | $\chi^2 = 2.67$ $P = 0.102$ | $\chi^2 = 6.41$ $P = 0.011$ | $\chi^2 = 8.09$ $P = 0.004$ | $\chi^2 = 7.17$ $P = 0.007$ |
| Moderately differentiated cases[a] ($n = 22$) | $\chi^2 = 2.79$ $P = 0.094$ | $\chi^2 = 1.47$ $P = 0.224$ | $\chi^2 = 4.77$ $P = 0.028$ | $\chi^2 = 5.86$ $P = 0.015$ |

[a]Gleason score 5–7.

the converse (Table 2). Nuclear localization of β-catenin could not be estimated in our samples because of the frozen sections and haematoxylin counterstaining.

Expression of E-cadherin and the three types of catenin was significantly correlated with histological differentiation (Table 3) but not lymphatic or vascular invasion (data not shown). Aberrant expression of the three types of catenin was associated with capsular invasion (Table 4). In moderately differentiated cases, aberrant expression of β- and γ-catenin was associated with capsular invasion, but that of E-cadherin and α-catenin was not (Table 4).

### DISCUSSION

Escape from primary nests as the initial step of cancer invasion requires disruption of the normal cell–cell adhesion in the epithelial tissue, which is dependent on the linkage between E-cadherin and the actin cytoskeleton via catenins (Kadowaki et al, 1994). Even if cadherin is present, intercellular adhesion is impaired if the connections are disturbed (Kadowaki et al, 1994). Thus, studying the patterns of both E-cadherin and catenins expression in cancer tissues with respect to invasion is warranted. The current study was the first, to our knowledge, to examine expression of E-cadherin and α, β, γ-catenin in prostate cancer using immunohistochemistry, although the relationship between E-cadherin and α-catenin expression has been investigated (Umbas et al, 1997; Richmond et al, 1997).

Our finding of association with differentiation is consistent with previous reports (Kadowaki et al, 1994; Matsui et al, 1994; Umbas et al, 1994; Rimm et al, 1995; Syrigos et al, 1995; Siitonen et al, 1996). The consistency in data for the four molecules is also in line with earlier results. It has been reported that α- and γ-catenin are unstable in L cells lacking cadherin, but that they can be stabilized by desmosomal cadherins and E-cadherin, respectively (Nagafuchi et al, 1991; Kowalczyk et al, 1994). Thus catenins expressed normally in 10 cases lacking E-cadherin might have been stabilized by other molecules. Aberrant expression of three types of catenin was also associated with capsular invasion, although the significant relationship was retained only for β- and γ-catenin when restricted to moderately differentiated (Gleason score 5–7) tumours. Of the four discrepant cases showing normal expression of E-cadherin and aberrant expression of at least one type of catenin, three (75%) demonstrated capsular invasion. Inclusion of these cases was likely to have affected the relationship. With regard to the lack of correlation with other types of invasion, only a few lesions were positive for the vascular type, making analysis difficult while conversely lymphatic invasion was frequent.

Interestingly, both E-cadherin and catenins were normally expressed in six of 22 cases of capsular invasion, 14 of 31 with lymphatic invasion and three of eight with vascular invasion. It has been suggested that abnormal tyrosine phosphorylation of β-catenin regulated by c-*erbB-2* protein or c-met protein may cause transient dysfunction of E-cadherin-mediated cell adhesion (Shibata et al, 1996). In prostate cancer, amplification and overexpression of c-*erbB-2* are rare (Pisters et al, 1995), but expression of c-met is frequently detectable immunohistochemically (Fournier et al, 1995). A possible explanation for the detachment of cancer cells from primary cancer nests in these cases is that abnormal tyrosine phosphorylation of β-catenin had occurred.

Reduced expression of E-cadherin and α-catenin appears to be associated with tumour progression and poor survival (Umbas et al, 1994, 1997; Richmond et al, 1997). Umbas et al (1994) have reported that progression after radical prostatectomy occurred in 67% (10 of 15) cases with aberrant E-cadherin expression, and in only 4% (one of 27) with normal E-cadherin staining. In the present study the period of observation is not sufficiently long (mean 18 months) to allow an assessment of prognosis. Long-term follow-up is continuing.

Our data indicate that three types of catenin may be useful predictive markers for biological aggressiveness of prostate cancer. We take a great interest in the question whether capsular invasion of prostate cancer can be more accurately predicted by immunostaining of catenins for biopsy materials before treatment. However, E-cadherin may be inadequate for this purpose despite the fact that this molecule has been regarded as a useful prognostic

 *© Cancer Research Campaign 1999*

marker. Actually it has been reported that half of the advanced prostate cancers with normal E-cadherin but aberrant α-catenin expression showed progression (Umbas et al, 1997). We have confirmed that formalin-fixed, paraffin-embedded specimens can be utilized for immunostaining of catenins (data not shown) and a prospective study using needle biopsy specimens is now in progress.

### REFERENCES

Behrens J, Mareel MM, Van Roy FM and Birchmeier W (1989) Dissecting tumor cell invasion: epithelial cells acquire invasive properties after the loss of uvomorulin-mediated cell–cell adhesion. *J Cell Biol* **108**: 2435–2447

Black RJ, Bray F, Ferlay J and Parkin DM (1997) Cancer incidence and mortality in the European Union: cancer registry data and estimates of national incidence for 1990. *Eur J Cancer* **33**: 1075–1107

Boring CC, Squires TS and Tong T (1993) Cancer statistics, 1993. *CA Cancer J Clin* **43**: 7–26

Bussemakers MJG, Van Moorselaar RJA, Giroldi LA, Ichikawa T, Issacs JT, Takeichi M, Debruyne FMJ and Schalken JA (1992) Decreased expression of E-cadherin in the progression of rat prostatic cancer. *Cancer Res* **52**: 2916–2922

Carter HB and Coffey DS (1989) Prediction of tumor behavior in prostate cancer. In *The Second Tokyo Symposium*. Karr JP, Yamanaka H (eds), pp. 19–27. Elsevier: New York

Fournier G, Latil A, Amet Y, Abalain JH, Volant A, Mangin P, Floch HH and Lidereau R (1995) Gene amplifications in advanced-stage human prostate cancer. *Urol Res* **22**: 343–347

Gleason DF (1977) Histological grading and clinical staging of prostatic carcinoma: urologic pathology. In *The Prostate*, Tannenbaum M (ed), pp 171–197. Lea and Fabiger: Philadelphia

Grayhack JT and Assimos DG (1983) Prognostic significance of tumor grade and stage in the patient with carcinoma of the prostate. *Prostate* **4**: 13–31

Hall RR (1996) Screening and early detection of prostate cancer will decrease morbidity and mortality from prostate cancer: the argument against. *Eur Urol Suppl* **2**: 24–26

Hinck L, Näthke IS, Papkoff J and Nelson WJ (1994) Dynamics of cadherin/catenin complex formation: novel protein interactions and pathways of complex assembly. *J Cell Biol* **125**: 1327–1340

Jawhari A, Jordan S, Poole S, Browne P, Pignatelli M and Farthing MJG (1997) Abnormal immunoreactivity of the E-cadherin-catenin complex in gastric carcinoma: relationship with patient survival. *Gastroenterology* **112**: 46–54

Kadowaki T, Shiozaki H, Inoue M, Tamura S, Oka H, Doki Y, Iihara K, Matsui S, Iwazawa T, Nagafuchi A, Tsukita S and Mori T (1994) E-cadherin and α-catenin expression in human esophageal cancer. *Cancer Res* **54**: 291–296

Korinek V, Barker N, Morin PJ, van Wichen D, de Weger R, Kinzler KW, Vogelstein B and Clevers H (1997) Constitutive transcriptional activation by a β-catenin-Tcf complex in APC–/– colon carcinoma. *Science* (Washington DC) **275**: 1784–1787

Kowalczyk AP, Palka HL, Luu HH, Nilles LA, Anderson JE, Wheelock MJ and Green KJ (1994) Posttranslational regulation of plakoglobin expression. *J Biol Chem* **269**: 31214–31223

Matsui S, Shiozaki H, Inoue M, Tamura S, Doki Y, Kadowaki T, Iwazawa T, Shimaya K, Nagafuchi A, Tsukita S and Mori T (1994) Immunohistochemical evaluation of alpha-catenin expression in human gastric cancer. *Virch Arch A* **424**: 375–381

Morin PJ, Sparks AB, Korinek V, Barker N, Clevers H, Vogelstein B and Kinzler KW (1997) Activation of β-catenin-Tcf signaling in colon cancer by mutations in β-catenin or APC. *Science* (Washington DC) **275**: 1787–1790

Nagafuchi A, Takeichi M and Tsukita S (1991) The 102 kd cadherin-associated protein: similarity to vinculin and posttranscriptional regulation of expression. *Cell* **65**: 849–857

Nakamura Y (1997) Cleaning up on β-catenin. *Nature Med* **3**: 499–500

Ochiai A, Akimoto S, Shimoyama Y, Nagafuchi A, Tsukita S and Hirohashi S (1994) Frequent loss of α catenin expression in scirrhous carcinomas with scattered cell growth. *Jpn J Cancer Res* **85**: 266–273

Ozawa M, Baribault H and Kemler R (1989) The cytoplasmic domain of the cell adhesion molecule uvomorulin associates with three independent proteins structurally related in different species. *EMBO J* **8**: 1711–1717

Pierceall WE, Woodard AS, Morrow JS, Rimm D and Fearon ER (1995) Frequent alterations in E-cadherin and α- and β-catenin expression in human breast cancer cell lines. *Oncogene* **11**: 1319–1326

Pisters LL, Troncoso P, Zhau HE, Li W, Eschenbach AC and Chung LWK (1995) C-met proto-oncogene expression in benign and malignant human prostate tissues. *J Urol* **154**: 293–298

Richmond PJM, Karayiannakis AJ, Nagafuchi A, Kaisary AV and Pignatelli M (1977) Aberrant E-cadherin and α-catenin expression in prostate cancer: correlation with patient survival. *Cancer Res* **57:** 3189–3193

Rimm DL, Sinard JH and Morrow JS (1995) Reduced α-catenin and E-cadherin expression in breast cancer. *Lab Invest* **72**: 506–512

Shibata T, Ochiai A, Kanai Y, Akimoto S, Gotoh M, Yasui N, Machinami R and Hirohashi S (1996) Dominant negative inhibition of the association between β-catenin and c-erbB-2 by N-terminally deleted β-catenin suppresses the invasion and metastasis of cancer cells. *Oncogene* **13**: 883–889

Shimazui T, Schalken JA, Giroldi LA, Jansen CFJ, Akaza H, Kenkichi K, Debruyne FMJ and Bringuier PP (1996) Prognostic value of cadherin-associated molecules (α-, β-, and γ-catenins and p120cas) in bladder tumors. *Cancer Res* **56**: 4154–4158

Siitonen SM, Kononen JT, Helin HJ, Rantala IS, Holli KA and Isola JJ (1996) Reduced E-cadherin expression is associated with invasiveness and unfavorable prognosis in breast cancer. *Am J Clin Pathol* **105**: 394–402

Smart CR (1997) The results of prostate carcinoma screening in the U.S. as reflected in the surveillance, epidemiology, and end results program. *Cancer* **80**: 1835–1844

Syrigos KN, Krausz T, Waxman J, Pandha H, Rowlinson-Busza G, Verne J, Epenetos AA and Pignatelli M (1995) E-cadherin expression in bladder cancer using formalin-fixed, paraffin-embedded tissues: correlation with histopathological grade, tumor stage and survival. *Int J Cancer* **64**: 367–370

Takeichi M (1991) Cadherin cell adhesion receptors as a morphogenetic regulator. *Science* (Washington DC) **251**: 1451–1455

Tominaga S and Kuroishi T (1997) An ecological study on diet/nutrition and cancer in Japan. *Int J Cancer Suppl* **10**: 2–6

Umbas R, Issacs WB, Bringuier PP, Schaafsma HE, Karthaus HFM, Oosterhof GO N, Debruyne FMJ and Schalken JA (1994) Decreased E-cadherin expression is associated with poor prognosis in patients with prostate cancer. *Cancer Res* **54**: 3929–3933

Umbas R, Issacs WB, Bringuier PP, Xue Y, Debruyne FMJ and Schalken JA (1997) Relation between aberrant α-catenin expression and loss of E-cadherin function in prostate cancer. *Int J Cancer* **74**: 374–377

Vleminckx K, Vakaet L Jr, Mareel M, Fiers W and Van Roy F (1991) Genetic manipulation of E-cadherin expression by epithelial tumor cells reveals an invasion suppressor role. *Cell* **66**: 107–119

Wasielewski R, Rhein A, Werner M, Scheumann GFW, Dralle H, Pötter E, Brabant G and Georgii A (1997) Immunohistochemical detection of E-cadherin in differentiated thyroid carcinomas correlates with clinical outcome. *Cancer Res* **57**: 2501–2507

*© Cancer Research Campaign 1999*
